# The growth hormone receptor interacts with transcriptional regulator HMGN1 upon GH-induced nuclear translocation

**DOI:** 10.1007/s12079-023-00741-2

**Published:** 2023-04-12

**Authors:** Lekha Jain, Mark H. Vickers, Bincy Jacob, Martin J. Middleditch, Daria A. Chudakova, Austen R. D. Ganley, Justin M. O’Sullivan, Jo K. Perry

**Affiliations:** 1 The Liggins Institute, University of Auckland, 85 Park Rd, Private Bag 92019, Auckland, 1142 New Zealand; 2 Faculty of Science, University of Auckland, Auckland, New Zealand; 3 School of Biological Sciences, University of Auckland, Auckland, New Zealand

**Keywords:** GHR, Nuclear, Mass spectrometry, Transcription factor, HMGN1

## Abstract

**Abstract:**

Growth hormone (GH) actions are mediated through binding to its cell-surface receptor, the GH receptor (GHR), with consequent activation of downstream signalling. However, nuclear GHR localisation has also been observed and is associated with increased cancer cell proliferation. Here we investigated the functional implications of nuclear translocation of the GHR in the human endometrial cancer cell-line, RL95-2, and human mammary epithelial cell-line, MCF-10A. We found that following GH treatment, the GHR rapidly translocates to the nucleus, with maximal localisation at 5–10 min. Combined immunoprecipitation-mass spectrometry analysis of RL95-2 whole cell lysates identified 40 novel GHR binding partners, including the transcriptional regulator, HMGN1. Moreover, microarray analysis demonstrated that the gene targets of HMGN1 were differentially expressed following GH treatment, and co-immunoprecipitation showed that HMGN1 associates with the GHR in the nucleus. Therefore, our results suggest that GHR nuclear translocation might mediate GH actions via interaction with chromatin factors that then drive changes in specific downstream transcriptional programs.

**Graphical abstract:**

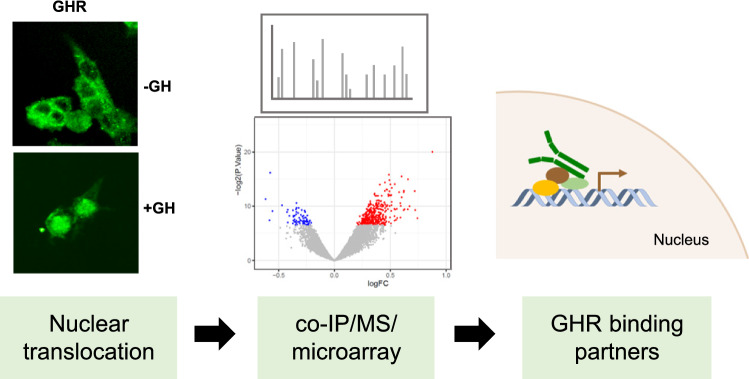

**Supplementary Information:**

The online version contains supplementary material available at 10.1007/s12079-023-00741-2.

## Introduction

The anterior pituitary hormone, growth hormone (GH), has wide-ranging effects on normal growth and metabolism with dysregulation of this hormone system occurring in many pathological conditions, including cancer (Chhabra et al. 2011; Perry et al. 2017). The GH receptor (GHR) belongs to the type I cytokine receptor family, a family of single-pass transmembrane receptors containing a minimum of one classic cytokine receptor homology domain. GHR activation following GH binding involves conformational changes in the receptor, leading to activation of associated non-receptor JAK2 and SRC family member tyrosine kinases, initiation of downstream signal transduction pathways (canonical GH signalling) (Carter-Su et al. 2016; Waters 2016; Dehkhoda et al. 2018; Lu et al. 2019), and altered gene transcription profiles (Perry et al. 2006; Rotwein and Chia 2010). GHR signalling is downregulated following GH binding by protein tyrosine phosphatases and members of the suppressors of cytokine signalling (SOCS) family, and the receptor is internalised by endocytosis and degraded in lysosomes (van Kerkhof et al. 2000; Strous and van Kerkhof 2002; Chhabra et al. 2021). GHR internalisation is facilitated by clathrin and/or caveolin systems (Lobie et al. 1999; Sachse et al. 2001; Yang et al. 2004). The GHR is also targeted for proteasome-dependent degradation by SOCS2-mediated ubiquitination (Greenhalgh et al. 2002).

In addition to traditional cell-surface GHR-mediated signalling, early literature observed nuclear localisation of GHR in certain tissues and demonstrated that the GHR is rapidly imported into the nucleus of cells upon stimulation with GH. This occurs in both normal cells and under pathological conditions, such as cancer (Lobie et al. 1992, 1994; Lincoln et al. 1998; Mertani et al. 1998; Conway-Campbell et al. 2007; Meng et al. 2022; Swanson and Kopchick 2007). The phenomenon has been shown to occur in cell lines from multiple species, including humans rodents, pigs and fish (Lobie et al. 1994; Mertani et al. 1998; Conway-Campbell et al. 2007; Figueiredo et al. 2016; Lan et al. 2017, 2018; Hainan et al. 2018). Lobie et al*.* reported that the full-length GHR is associated with the nucleus and that the intracellular domain was required for nuclear localisation (Lobie et al. 1994). This is in line with other studies demonstrating that numerous other classical cell-surface receptors that were previously thought to signal exclusively at the cell-surface can also translocate from the cell membrane to the nucleus upon stimulation, with some shown to directly influence gene expression. For example, the epidermal growth factor receptor (EGFR), which is also involved in tumorigenesis, translocates to the nucleus and functions as a transcription factor for multiple genes (Brand et al. 2011; Carpenter and Liao 2013; Shah et al. 2019). Other notable examples include the insulin receptor, fibroblast growth factor receptor, platelet-derived growth factor receptor beta and the IGF1 receptor (Aleksic et al. 2018; Shah et al. 2019; Werner et al. 2021).

Nuclear localisation of the GHR has functional consequences such as increased proliferative status in vitro and in vivo as demonstrated in cell lines, a liver regeneration model and in transgenic zebrafish models (Conway-Campbell et al. 2007; Figueiredo et al. 2016; Zheng et al. 2022). Furthermore, constitutive nuclear localisation of the GHR in murine pro-B cells resulted in metastatic tumours when injected into nude mice suggesting a role in tumorigenesis and tumour progression (Conway-Campbell et al. 2007). However, whether there are transcriptional changes associated with nuclear GHR import remains to be determined.

In this study we investigated whether the GHR interacts with proteins in the nucleus following nuclear translocation. This was coupled with transcriptional profiling to demonstrate that the transcriptional regulator, high mobility group nucleosome binding domain 1 (HMGN1), is a novel nuclear binding partner of the GHR, with corresponding changes in expression of HMGN1 target genes observed following GH treatment. Therefore, this study identifies novel targets of GHR signalling and provides a potential mechanism through which nuclear import of GHR can act in parallel to the canonical signal transduction pathway to mediate the effects of GH.

## Materials and methods

### Cell lines and reagents

Cell lines were purchased from the American Type Culture Collection. The human endometrial carcinoma cell-line RL95-2 was maintained in RPMI-1640 growth medium supplemented with 5% fetal bovine serum, 100 μg/ml streptomycin, 100 U/ml penicillin, and Glutamax. The human mammary epithelial cell-line MCF-10A was maintained in MEGM™ Mammary Epithelial Cell Growth Medium BulletKitTM (Lonza) supplemented with 100 μg/ml streptomycin, 100 U/ml penicillin, and 5% normal horse serum. Cells were cultured in a humidified chamber with 5% CO_2_ at 37 °C. Cells in the logarithmic phase of growth were used for hormone treatment, western blot, mass spectrometry, and microarray analyses.

Recombinant GH was purchased from the National Hormone and Pituitary Program (NHPP) and reconstituted in sterile phosphate-buffered saline (PBS, pH 7.4). Antibodies used in the experiments were GHR extracellular domain (Abcam, ab89400) GHR intracellular domain (Santa Cruz Biotechnology, sc-137185), HMGN1 (Invitrogen Life Technologies, 720,387), SUMO1 (Invitrogen Life Technologies, 332,400), anti-HDAC1 (Abcam, ab19845) phospho-STAT5 (pTyr694) (Life Technologies; 71–6900), STAT5 (C-17) antibody (Santa Cruz Biotechnology; sc-835), β-actin (Sigma-Aldrich;A1978); goat anti-mouse IgG (Sigma-Aldrich, A4416), goat anti-mouse Alexa Fluor 488 (Invitrogen, A-11001) and goat anti-rabbit IgG (Sigma-Aldrich, A3687) secondary antibodies; and isotype control antibody (ThermoFisher Scientific, 31,903).

## Western blotting

Cells were plated in T75 cell culture flasks at 5 × 10^6^ cells per flask, serum-starved overnight, then treated with GH at different concentrations (0, 50, 100, 250 and 500 ng/ml). For whole cell lysates, cells were lysed in 50 mM Tris–HCL (pH 7.4),1% Nonidet P-40; 150 mM NaCl, 1 mM EDTA, 1 mM NaF, 1 mM phenylmethylsulfonylfluoride, 1 mM Na_3_VO_4_, and protease inhibitor cocktail (Life Technologies). For subcellular fractionation, cytoplasmic and nuclear fractions were extracted using the NE-PER fractionation kit (Thermo Fisher Scientific), according to the manufacturer’s instructions.

Proteins were resolved by SDS-PAGE (12% resolving gel) and transferred to nitrocellulose membranes which were blocked with 5% BSA for 1–2 h at room temperature. After washing with PBS-T (PBS + 0.1% Tween 20) solution, membranes were incubated overnight at 4 °C, with the primary antibodies as indicated. Membranes were subjected to three PBS-T washes, followed by incubation with a horseradish peroxidase–conjugated secondary antibody for 1 h at room temperature. Membranes were then washed three times, and proteins visualised using Clarity Western Peroxide Reagent (Bio-Rad) and a Bio-Rad Chemidoc MP system.

## Immunofluorescence

RL95-2 or MCF-10A cells (2 × 10^4^) were plated on coverslips in 6-well plates and serum-starved overnight. Following stimulation with GH at the indicated times and concentrations, cells were fixed with 4% paraformaldehyde (w/v) at room temperature for 10 min. Fixed cells were washed with PBS three times and permeabilised with 0.5% Triton X-100 for 30 min. After washing, the coverslips were blocked with 5% BSA in PBS (pH 7.4) for 2 h at room temperature, washed with PBS and incubated with primary or mouse IgG control antibodies overnight at 4 °C, followed by secondary antibodies for 1 h at 37 °C. Coverslips were washed, stained, and mounted using SlowFade™ Diamond Antifade Mountant with DAPI (Thermo Fisher Scientific). Cells were visualised using confocal laser scanning microscopy (Zeiss LSM 800 Airyscan confocal microscope) with × 63 oil immersion objectives. Image analysis was performed using ZEN Blue and ImageJ software.

## Immunoprecipitation

RL95-2 cells were plated in T-175 cell culture flasks with 5 × 10^6^ cells per flask and were serum-starved overnight. Following GH treatment (500 ng/ml) for 10 min, cells were either lysed to prepare whole-cell lysates (for mass spectrometry) or fractionated into nuclear and cytosolic lysates (method described above). Cell lysates were pre-cleared with magnetic protein-G conjugated beads. Pre-cleared lysates (1 mg/ml) were incubated with 10 µg of primary antibody or isotype control antibody overnight at 4 °C on a rotating wheel. 100 µL of protein-G conjugated beads were then incubated with lysate-antibody complexes at 4 °C for 2–4 h with rotation. The magnetic beads were separated using a magnetic rack, the solution was discarded, and the beads washed 5–8 times with PBS pH 7.4 to reduce non-specific binding. Immunoprecipitated proteins were detected by western blotting under reducing conditions, as described above, or by mass spectrometry. For mass spectrometry, protein lysates for each replicate (n = 3 per group) were diluted to a concentration of 1 mg/ml.

## Mass spectrometry

Following immunoprecipitation, beads were washed twice with freshly prepared 100 mM ammonium hydrogen carbonate (AMBIC) solution. Protein complexes bound to the washed beads were eluted by incubation with 100 µl of 5% acetic acid for 2 min. Following this, the eluate was concentrated using a centrifugal vacuum concentrator, and diluted with bicarbonate solution to pH 8. This sample was subjected to reduction with dithiothreitol, alkylation with iodoacetamide, and digestion with 0.5 µg sequencing grade modified porcine trypsin (Promega, Madison, WI, USA). The digest was then desalted on HLB cartridges (Waters, Milford, MA, USA) by solid-phase extraction (SPE), and concentrated to a volume of ~ 15 µl using a centrifugal vacuum concentrator.

Samples were injected on a 0.3 × 10 mm trap column packed with 3u Reprosil C18 media (Dr Maisch), and desalted for 5 min at 10 µl/min, prior to separation on a 0.075 × 200 mm picofrit column (New Objective) that was packed in-house with 3u Reprosil C18 medium. A gradient was then applied at 300 nl/min using a Exsigent NanoLC 400 UPLC system (Sciex): 0 min 5% B; 45 min 40% B; 47 min 95% B; 50 min 95% B; 50.5 min 5% B; 60 min 5% B, where A was 0.1% formic acid in water and B was 0.1% formic acid in acetonitrile.

The picofrit spray was directed into a TripleTOF 6600 Quadrupole-Time-of-Flight mass spectrometer (Sciex) scanning from 350 to 2000 m/z for 200 ms, followed by 45 ms MS/MS scans on the 40 most abundant multiply-charged peptides (m/z 80–1600) for a total cycle time of ~ 1.8 s. The mass spectrometer and HPLC system were under the control of the Analyst TF 1.7 software package (Sciex).

## Mass spectrometry data analysis

The mass spectrometry data was searched against a database comprising Uniprot Human entries appended with a set of common contaminant sequences, using ProteinPilot version 5.0 (Sciex). Search parameters were: Sample Type, Identification; Search Effort, Thorough; Cys Alkylation, Iodoacetamide; Digestion, Trypsin. The peptide summary was exported from ProteinPilot and underwent further processing in Excel. Proteins with Unused Scores below 1.3 were removed, inferior or redundant peptide spectral matches were eliminated, and intensities for all unique peptides from each protein were summed. Following this, peptides present in even one replicate of IgG control samples were filtered out from the GH untreated and treated datasets. In addition, proteins that were not present in all three replicates were excluded.

The processed data were loaded into an MSstats R package (Choi et al. 2014) for normalisation and differential expression analysis. Normalisation was performed to remove systematic bias between MS runs using the default ‘equalizeMedians’, which represents constant normalisation (equalising the medians) based on reference signals. This was followed by summarisation of the data using a Tukey Median Polish algorithm, followed by differential expression analysis between the control and treatment samples. *P* values were adjusted using the Benjamini–Hochberg correction method (Benjamini and Hochberg 1995). Pathway analysis was performed using g:Profiler package in R (Raudvere et al. 2019).

## RNA extraction, reverse transcriptase PCR (RT-PCR), and microarray

RL95-2 or MCF-10A cells were plated in 10 cm cell culture dishes at 5 × 10^6^ cells per dish, serum-starved overnight, then treated with 500 ng/mL GH for 90 min. Total RNA was extracted using TRIzol reagent (Life Technologies), and column-purified using the RNeasy mini kit (Qiagen) according to the manufacturer’s instructions. The purity and concentration of extracted RNA were determined using a NanoDrop ND-1000 instrument (Thermo Fisher Scientific). The integrity of extracted RNA was assessed by Bioanalyser using an RNA 6000 Nano LabChip kit according to the manufacturer’s instructions (Agilent). RNA integrity numbers (RINs) were all greater than 9.

Microarray analysis was performed by Auckland Genomics (University of Auckland), using ClariomD microarray (Thermo Fisher Scientific). Sample labelling, microarray hybridisation, and washing were performed according to the manufacturer’s instructions. The array images were acquired by means of the Affymetrix GeneChip Operating Software.

For RT-PCR, total RNA was DNAse I treated and 500 ng was used for cDNA synthesis (High-Capacity cDNA Reverse Transcription Kit, ThermoFisher) with 1 μl used as template. Primer sequences for amplification were: human prolactin receptor (PRLR) (5'-CGCGAAACAGCTTTCCACAC and 5’-CAGATGCCACATTTTCCTTC), human GHR (5’-CTCAACTGGACTTTACTGAACG and 5’-AATCTTTGGAACTGGAACTGGG), and β-ACTIN (5’-ATGATATCGCCGCGCTCG and 5’-CGCTCGGTGAGGATCTTCA).

## Microarray data analysis

Data normalisation and subsequent data processing were performed in R (R version 3.4). Differentially expressed genes at each time point were estimated using the R packages maEndToEnd (Klaus and Reisenauer 2016) and limma (Ritchie et al. 2015). Transcripts with *P*-value < 0.01 were considered significantly differentially expressed. Differentially expressed genes were normalised at the transcript level using the robust multi-array average method. Volcano plots for representation of differentially expressed genes were generated using the ggplot2 R package (Gómez-Rubio 2017). Pathway analysis of differentially expressed genes was performed with the g:Profiler package in R (Raudvere et al. 2019).

## Statistical analysis

All normally distributed data are expressed as means ± S.E.M and were compared using one-way ANOVA with post-hoc analysis (Tukey's procedure) as appropriate. Unless otherwise stated, assays were repeated at least three times with a representative figure shown. A p-value of < 0.05 was accepted as statistically significant.

## Results

### The GHR translocates into the nucleus following stimulation with GH

**Fig. 1:**
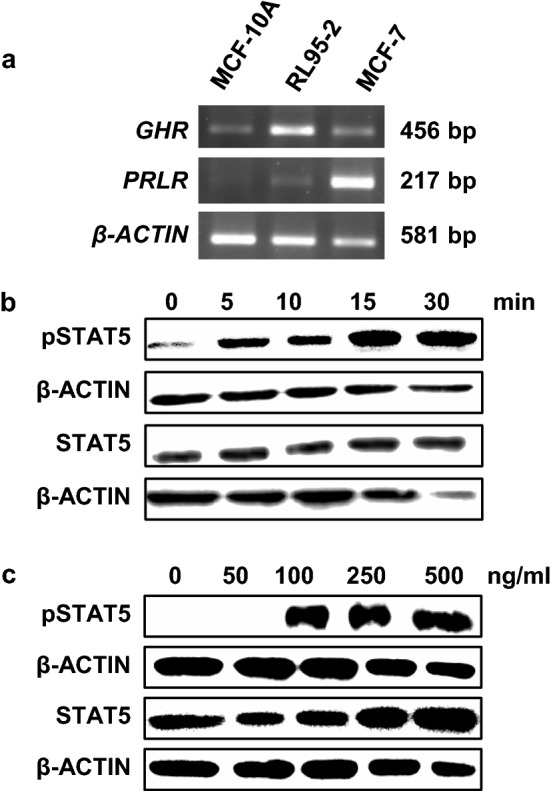
Characterisation of GH response in RL95-2 cells. **a** Semi-quantitative RT-PCR analysis of *GHR* and *PRLR* mRNA expression in RL95-2, MCF-10A, and the breast cancer cell line MCF7 (positive control). **b** GH treatment time-course in RL95-2 cells. Serum-starved RL95-2 cells were treated with 500 ng/ml recombinant human GH for 0, 5, 10, 15 and 30 min. **c** GH treatment concentration–response in RL95-2 cells. Serum-starved RL95-2 cells were treated with 0, 50, 100, 250 and 500 ng/ml GH for 15 min. Cell lysates were immunoblotted for phosphorylated STAT5 (pSTAT5) and total STAT. β-ACTIN was used as a loading control for all experiments

To investigate the effects of nuclear translocation of the GHR, we first tested whether GHR nuclear translocation is observed in the endometrial cancer cell-line, RL95-2, and the mammary epithelial cell-line, MCF-10A. Given that GH can also activate a closely related type I cytokine receptor, the prolactin receptor (PRLR), PRLR expression was also investigated (Goffin et al. 1996). The breast cancer cell line, MCF-7, which is known to express *GHR* and *PRLR* mRNA (Cancer Cell Line Encyclopedia) was used as a positive control. *GHR* mRNA expression was confirmed in both cell lines by semi-quantitative RT-PCR, with higher expression observed in RL95-2 (Fig. 1a). Both RL95-2 and MCF-10A cell lines expressed very low levels of *PRLR* mRNA compared to MCF-7 (Fig. 1a).

To confirm that RL95-2 and MCF-10A cells responded to GH, GH-dependent phosphorylation of the downstream signal transduction molecule, signal transducer and activator of transcription 5 (STAT5), was measured by western blotting at 0, 5, 10, 15, and 30 min post-treatment, and in response to different GH concentrations (0, 50, 100, 250 and 500 ng/ml). GH treatment increased STAT5 phosphorylation in RL95-2 and MCF-10A cells (Fig. 1b, c, and Supplementary Fig. 1), with maximal stimulation after 15 min observed at a concentration of 500 ng/ml GH in RL95-2 cells (Fig. 1c) and 250 ng/ml in MCF-10A cells (Supplementary Fig. 1). Thus, both cell lines exhibited a robust response to GH.

**Fig. 2:**
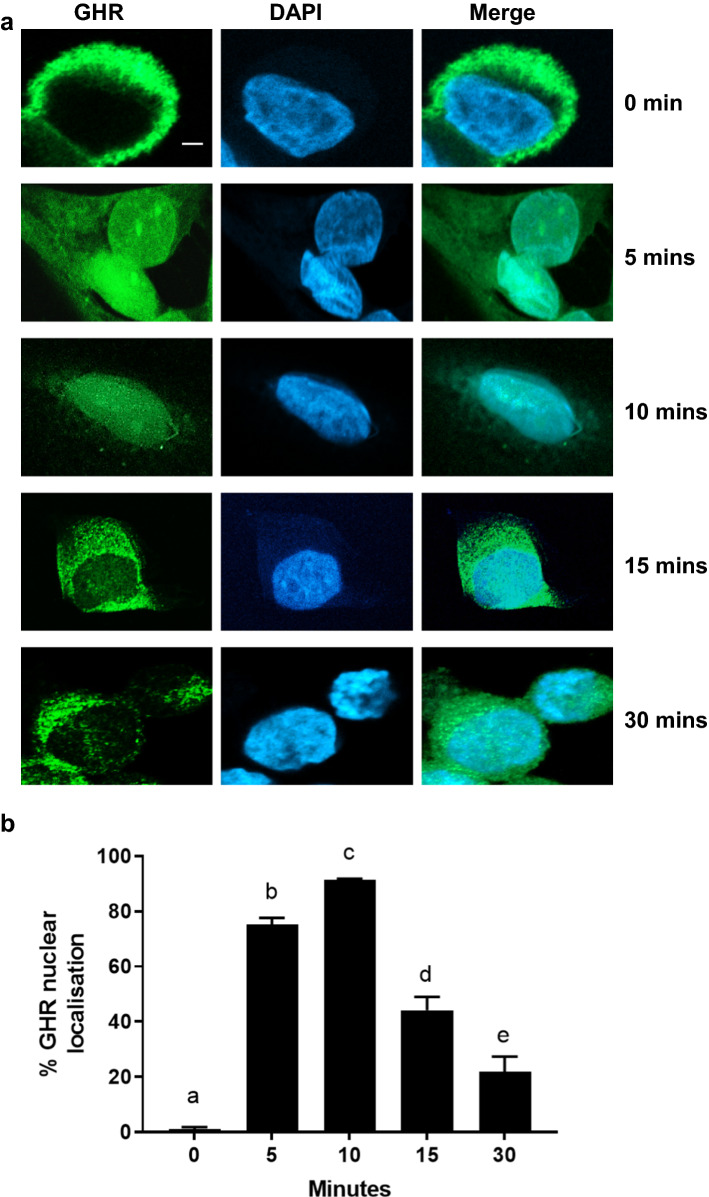
Subcellular localisation of GHR in RL95-2 cells following GH treatment. **a** RL95-2 cells were grown on coverslips, serum-starved and treated with 500 ng/ml recombinant human GH for 0, 5, 10, 15 and 30 min. Cells were then fixed, permeabilised, blocked and immuno-stained with anti-GHR antibody and fluorescent secondary antibody. The slides were visualised using confocal laser scanning microscopy. Green (Alexa-Fluor 488) represents GHR staining (anti-GHR_IC_ antibody, sc-137185) and blue (DAPI) is a nuclear stain. Scale bar is 100 µm. **b** Quantification of cells with nuclear GHR localisation at different time points. Data are presented as mean ± SEM (*n* > 100 per timepoint). Groups with different letters are significantly different from each other (*P* < 0.001, One-way ANOVA). This experiment was repeated at least three times and a representative figure is shown

We next determined if the GHR translocates to the nucleus in RL95-2 and MCF-10A cells. Immunofluorescence consistent with nuclear GHR localisation was observed in RL95-2 cells 5 min after treatment with 500 ng/ml GH using a GHR antibody that targets the extracellular domain of the GHR (anti-GHR_EC_). Maximal localisation was observed at 10 min, with a significant decline at 15 min (Fig. 2a, b). A similar trend was observed for MCF-10A cells (Supplementary Fig. 2). Nuclear localisation of the GHR was also detected in both cell lines by immunofluorescence using a separate anti-GHR antibody that targets the intracellular domain of the GHR (anti-GHR_IC_) (Supplementary Fig. 3). Immunoprecipitation and western blot analysis using both anti-GHR antibodies also detected GHR in nuclear extracts following GH treatment (Supplementary Fig. 4).

Collectively, these results indicate that the GHR rapidly translocates to the nucleus in RL95-2 and MCF-10A cells. RL95-2 was used in subsequent experiments due to the higher levels of GHR expression that were observed in this cell-line.

## Proteomic analysis of proteins that co-immunoprecipitate with the GHR

**Fig. 3:**
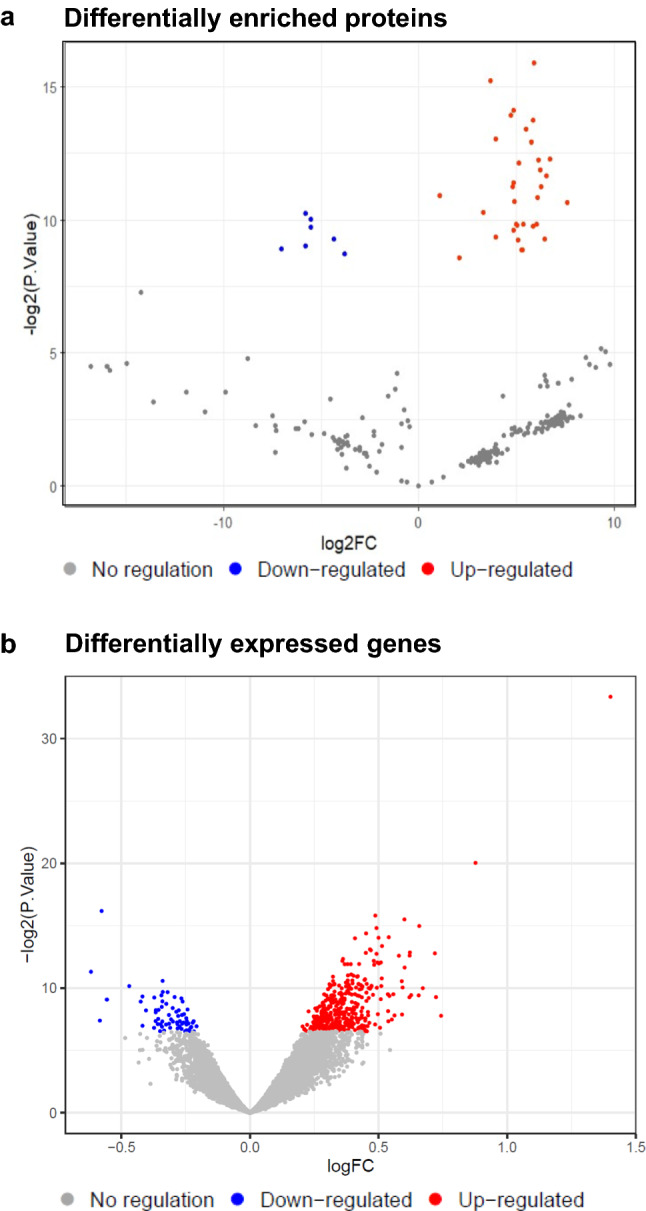
**a** Volcano plot showing proteins identified by co-immunoprecipitation with GHR coupled with mass spectrometry that were differentially enriched following treatment with 500 ng/ml GH for 5 min, compared to control (log2FC). Blue dots represent proteins significantly depleted in treated samples compared to the control; red dots proteins either significantly enriched compared to the control following GH treatment or only present in the GH-treated group. **b** Volcano plot representation of differentially expressed genes between control and treatment with 500 ng/ml GH for 90 min (log2FC) obtained using a Clariom D microarray. Blue dots represent significantly downregulated genes (61 genes) and red dots upregulated genes (355 genes)

Next, to help determine the significance of the nuclear re-localisation of the GHR, we sought to identify novel GHR binding partners. To achieve this, co-immunoprecipitation by anti-GHR antibodies coupled with mass spectrometry was performed on total cell lysates from RL95-2 cells treated with or without GH. For mass spectrometry, total cell lysates were split in two and immunoprecipitated separately with the anti-GHR_IC_ and anti-GHR_EC_ antibodies, then eluates were combined and processed for mass spectrometry. Anti-mouse IgG was used as an immunoprecipitation control. Initial analysis confirmed the presence of the GHR in lysates immunoprecipitated with the anti-GHR antibodies (data not shown). Further analysis of the mass spectrometry data identified that the levels of 40 proteins were found to be significantly different in GHR-immunoprecipitated lysates from GH treated versus control cells (P value < 0.05, FDR < 0.05) (Table 1; Supplementary Table 1; Fig. 3a), of which, 32 were enriched in lysates from GH-treated cells and 8 were depleted. Fifteen proteins were found to be present in GH-treated but not control cells, suggesting that these proteins only interact with the GHR after GH treatment (Supplementary Tables 2–4).

**Table 1:** Proteins identified by GHR co-immunoprecipitation and mass spectrometry analysis to be differentially enriched between control and GH treated samples (FDR < 0.05)

Protein	log2FC	SE	FDR	Function
AK2	5.90	0.02	0.01	Catalyses reversible transfer of terminal phosphate group between ATP and AMP
PSMD6	3.69	0.02	0.01	Component of the 26S proteasome, protein homeostasis
LAMC1	4.74	0.04	0.01	Laminin subunit, extracellular matrix
RCC2	4.85	0.04	0.01	Guanine exchange factor, kinetochore-microtubule function in early mitosis
EIF5A	5.86	0.05	0.01	mRNA-binding protein involved in translation elongation
HNRNPU	5.49	0.05	0.01	DNA and RNA binding, nuclear chromatin organisation, pre-mRNA processing
CAD	3.94	0.04	0.01	Enzyme involved in pyrimidine biosynthesis
SUMO1	5.76	0.07	0.01	Ubiquitin-like protein, nuclear transport, DNA replication/repair, mitosis, signalling
ACO2	5.11	0.08	0.01	TCA cycle enzyme, catalyses the isomerisation of citrate to isocitrate
TMPO	6.14	0.09	0.01	Lamina-associated polypeptide, inner nuclear membrane protein
LASP1	6.72	0.09	0.01	Actin-binding protein, cytoskeletal organisation
EEF1A1	6.24	0.10	0.01	Eukaryotic translation elongation factor 1 alpha-1
PLIN3	4.83	0.10	0.01	Mannose 6-phosphate receptor transport
CRIP2	4.87	0.09	0.01	Zinc ion binding protein, putative transcription factor smooth muscle
CALM3	6.27	0.13	0.01	Calcium-binding protein, regulation of cell cycle and cytokinesis
RCN1	6.55	0.12	0.01	Calcium-binding protein in the endoplasmic reticulum lumen
ATP1A1	1.09	0.02	0.02	Na + /K + cation transport ATPase
EEF1D	6.11	0.14	0.02	Subunit of the elongation factor-1 complex
DDX21	4.92	0.12	0.02	RNA helicase
MYL6	7.58	0.19	0.02	Regulatory light chain of myosin
RPS18	− 5.80	0.17	0.02	Ribosomal protein, component of the 40S subunit
TES	3.32	0.09	0.02	Cell adhesion and actin reorganisation
ITIH3	− 5.53	0.17	0.02	Heavy chain subunit of the pre-alpha-trypsin inhibitor complex
RPN1	5.00	0.17	0.02	Subunit of the N-oligosaccharyl transferase complex
FLNA	5.03	0.17	0.02	Actin-binding protein that crosslinks actin filaments
SHTN1	5.34	0.18	0.02	Neuronal polarization and neurite outgrowth
SLC1A5	5.88	0.20	0.02	Sodium-dependent amino acid transporter
PFDN2	6.05	0.20	0.02	Subunit of prefoldin, a molecular chaperone complex
HSPA8	− 5.54	0.19	0.03	Heat shock protein 70 family chaperone protein
HMGN1	4.87	0.18	0.03	Binds nucleosomal DNA, associated with transcriptionally active chromatin
USP5	3.94	0.15	0.03	Ubiquitin proteinase
EEF1G	− 4.34	0.17	0.03	Eukaryotic Translation Elongation Factor 1 Gamma
TCOF1	5.11	0.21	0.03	Nucleolar protein
RPL35	6.48	0.26	0.03	Ribosomal protein, component of the 60S subunit
ALB	− 5.83	0.26	0.04	Serum albumin protein
RPS12	− 7.04	0.32	0.04	Ribosomal protein, component of the 40S subunit
EGFR	5.25	0.24	0.04	Epidermal growth factor receptor
PFDN6	5.34	0.25	0.04	Subunit of prefoldin, a molecular chaperone complex
HSPA4	− 3.82	0.19	0.04	Heat shock protein 70 family chaperone protein
PHGDH	2.07	0.11	0.04	Phosphoglycerate Dehydrogenase, metabolic protein

Pathway analysis was conducted using g: Profiler to determine if proteins found in complex with the GHR were enriched in specific biological pathways. This analysis demonstrated that the majority of these proteins were enriched in known cellular processes, such as translation of proteins, ribosomal pathways, cytoskeletal organisation and trafficking, cell-cycle maintenance, splicing, ubiquitination, and metabolism (Supplementary Tables 5–7). This analysis therefore identified potential new GHR binding partners. Of particular interest were transcriptional regulators that may be mediating transcriptional regulation by GH in the nucleus, therefore we focused on these.

## Transcriptional regulator, HMGN1, associates with the GHR in the nucleus and GH increases expression of its gene targets

Translocation of the GHR to the nucleus upon GH treatment suggests that it may play a role in the nucleus linked to the regulation of gene expression, as has been shown for other cell-surface receptors translocating to the nucleus. To look for evidence of this, we used the ChIP-Atlas database (Oki et al. 2018) to investigate whether any of the proteins identified by mass spectrometry were transcription regulators. We found that three of the proteins enriched for interaction with the GHR upon GH treatment were transcriptional regulators, HMGN1, SUMO1, and DDX21. The ChIP-Atlas database indicated that these have 1140, 4354, and 9674 putative gene targets, respectively. If the association between these regulators and the GHR is functionally relevant, GH treatment would be predicted to induce changes in expression of their target genes. To test this, we used microarray analysis to determine whether targets of HMGN1, SUMO1, and DDX21 are regulated by GH in RL95-2 cells. RL95-2 cells were treated with or without 500 ng/mL GH for 90 min, and gene expression was analysed using Clariom D microarrays. We identified 416 genes that were differentially expressed (DE) following GH stimulation (Fig. 3b, Supplementary Table 8). Pathway analysis demonstrated that these genes were significantly enriched in pathways such as VEGF/VEGFR, mRNA processing, TGF-β, adipogenesis, nuclear receptor, and EGF/EGFR signalling (Supplementary Tables 9–11).

Initially we investigated whether any of the DE genes were targets of STAT5, which is a transcription factor activated by GHR signalling. To identify GH-regulated genes that may be transcriptionally regulated via activation of STAT5, the list of identified DE genes was intersected with 7418 known STAT5 (STAT5A and STAT5B) gene targets (ChIP-Atlas database). This analysis demonstrated that 194 of the 416 genes that were DE in RL95-2 cells following GH stimulation were targets of STAT5 (Supplementary Table 12). Next, we intersected known gene targets of the three transcription regulators identified by co-immunoprecipitation mass spectrometry analysis (i.e. HMGN1, SUMO1, and DDX21) with the list of GH-regulated DE genes. Of these, 43 of the DE genes were HMGN1 targets, 124 genes were SUMO1 targets, and 253 of the DE genes were DDX21 targets (Supplementary Table 13). Bootstrapping of these values indicated that the number of genes is not likely to be aleatory for any of these transcription regulators (*P* value < 0.001), suggesting that GH treatment alters the transcriptional profile of STAT5, HMGN1, SUMO1, and DDX21 target genes.

**Fig. 4:**
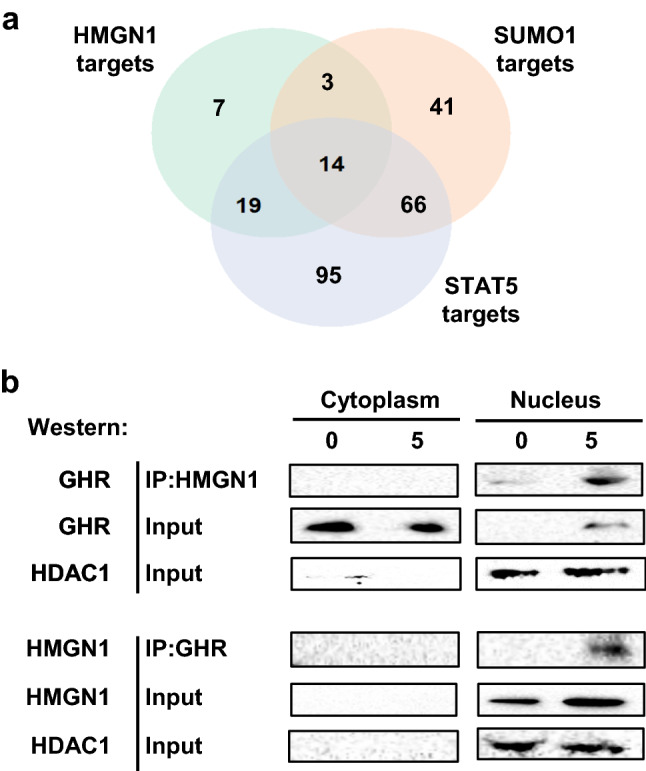
**a** Venn diagram showing the intersection of known gene targets of HMGN1, SUMO1, and STAT5 obtained from the ChIP-atlas database with differentially expressed genes from microarray analysis. **b** Investigation of nuclear association of the GHR with HMGN1 by co-immunoprecipitation followed by western blotting. Serum-starved RL95-2 cells were treated with 500 ng/ml recombinant human GH for 0 and 5 min, lysed, and cytoplasmic and nuclear fractions isolated. Proteins were immunoprecipitated with the combination of extracellular and intracellular (ab89400 and sc-137185) anti-GHR antibodies or an HMGN1 antibody. Eluates were separated by SDS-PAGE and immunoblotted using antibodies to HMGN1 or GHR (ab89400) as indicated. HDAC1 was used as a nuclear control

To look for independent evidence of transcriptional changes mediated by GHR interaction with these transcriptional regulators, we used the RegulatorTrail database, which identifies transcriptional regulators from transcriptomic and epigenomic data (Kehl et al. 2017), to predict potential transcription factors from the gene expression dataset. RegulatorTrail analysis of the RL95-2 DE genes identified both STAT5A and 5B as transcription regulators (Supplementary Table 14). The only two proteins from the mass spectrometry data present on the list of regulators generated by RegulatorTrail were HMGN1 and SUMO1 (Supplementary Table 14). Therefore, complementary evidence suggests that STAT5, HMGN1 and SUMO1 are involved in the altered gene expression profiles observed upon GH treatment, and their interaction with GHR further suggests that GHR translocation to the nucleus may be an important factor in this transcriptional response. Seventeen genes were shared between HMGN1 and SUMO1 targets (Fig. 4a). Of the STAT5 genes targets, 33/194 were in common with HMGN1 targets and 81/194 were in common with SUMO1 targets (Supplementary Table 15). In addition, fourteen genes were targets of all three regulators (HMGN1, SUMO1 and STAT5).

Given that stronger supporting evidence was available for HMGN1 and SUMO, we next attempted to validate the interaction between the GHR and these proteins by co-immunoprecipitation/western blot. HMGN1 was of particular interest as the related type I cytokine receptor, PRLR, has previously been shown to bind to another high-mobility group protein, HMGN2, following nuclear localisation and may facilitate transcriptional activation by STAT5A (Fiorillo et al. 2011). As the co-immunoprecipitation/mass spectrometry was performed on whole cell lysates, a second co-immunoprecipitation and western blot experiment was performed on cells that had been fractionated in to cytoplasmic and nuclear fractions to look for direct evidence of HMGN1 and/or SUMO1 interactions with the GHR in the nucleus. HMGN1 was localised exclusively in the nucleus of RL95-2 cells, and was found to be associated with the GHR following 5 min of GH treatment, as demonstrated by co-immunoprecipitation using the combination of anti-GHR_IC_ and anti-GHR_EC_ antibodies as bait (Fig. 4b). Similar results were observed when using the anti-HMGN1 antibody as a bait, again with the association being very strongly stimulated by GH treatment as expected (Fig. 4b). Immunoprecipitation with a non-specific IgG was used as a control (data not shown). SUMO1 was detected in the cytoplasm and nucleus, but no association between SUMO1 and GHR was observed in either anti-SUMO1 or anti-GHR immunoprecipitations (data not shown). It is unclear why we could detect association between the GHR and SUMO1 in the mass-spec analysis but not here. This could be attributed to the additional processing required for subcellular fractionation, particularly if the interaction is weak. Therefore, an association between the GHR and SUMO in the nucleus remains to be confirmed. Collectively, these results suggest that the GHR and HMGN1 are localised in a complex in the nucleus that is involved in GH-dependent coordinated regulation of expression of a subset of HMGN1 gene targets.

## Discussion

Here, we show that the GHR rapidly translocates into the nucleus in RL95-2 cells following stimulation by GH, and identify transcriptional regulator, HMGN1, as a novel binding partner of the GHR in the nucleus. This co-localisation correlates with changes in expression of a subset of known gene targets of HMGN1. Our results are interesting in light of the growing recognition of the role of nuclear localisation of cell receptors, as nuclear translocation is not unique to the GHR. Over the past few decades it has been demonstrated that multiple cell-surface receptors, previously believed to exclusively signal at the cell-surface, localise to the nucleus following a suitable stimulus (Lobie et al. 1994; Bryant and Stow 2005; Conway-Campbell et al. 2007; Wang and Hung 2009), and in some cases have been shown to result in transcriptional changes. Several studies have previously demonstrated the presence of the GHR in the cell nuclei of cell lines from different species, with translocation often occurring within 5 to 10 min following GH stimulation (Lobie et al. 1994; Conway-Campbell et al. 2007; Figueiredo et al. 2016; Lan et al. 2017). Consistent with these studies, rapid nuclear translocation of the GHR was observed in RL95-2 and MCF-10A cells within 5 min of GH treatment, with maximal localisation at 10 min.

Immunoprecipitation using anti-GHR antibodies, combined with mass spectrometry, identified 40 protein-binding partners of the GHR in RL95-2 cells. Pathway analysis identified an enrichment in eukaryotic translation, ribosomes, cellular trafficking, cell-cycle, and splicing pathways. There was also a GH-induced association of the GHR with proteins involved in regulation of the cytoskeleton and membrane trafficking. Mass spectrometry analysis also identified increased association of the GHR with proteins involved in ubiquitination following GH treatment.

GHR nuclear localisation suggests a role in transcriptional regulation. In support of this hypothesis we found that the GHR associates with two transcriptional regulators, HMGN1 and SUMO1, in response to GH treatment. In particular, our results demonstrate an interaction between the GHR and HMGN1, which has not been reported previously. This association was confirmed by co-immunoprecipitation experiments in nuclear extracts and was supported by GH-dependent changes in the expression of putative HMGN1 gene targets. HMGN1 belongs to a family of high-mobility group nucleosome binding (HMGN) proteins which function as transcription regulators by binding to nucleosomes and altering DNA helix:histone interactions. HMGN1 specifically binds to histone H3, causing changes in its phosphorylation and acetylation (Lim et al. 2004, 2005) that affect gene expression by altering chromatin structure (Bannister and Kouzarides 2011; Kugler et al. 2013; Mowery et al. 2018). Further support for an interaction between GHR and HMGN1 comes from the observation that the related cytokine receptor, PRLR, interacts with another high-mobility group protein, HMGN2, following prolactin-induced nuclear localisation of the receptor and facilitates transcriptional activation by STAT5A (Fiorillo et al. 2011). Thus this class of transcriptional regulator may play a wider role in mediating GH and prolactin-stimulated transcription.

Although our results also identified SUMO1 as a potential GHR binding partner, interaction with SUMO1 is more equivocal, as the interaction identified by mass spectrometry was not confirmed by co-immunoprecipitation experiments. This might indicate that GHR indirectly activates SUMO1 by interaction with other proteins present in the same complex, or in close proximity. Some association between GH and SUMO1 is nevertheless suggested by our observation that genes changing expression upon GH treatment are strongly enriched for SUMO1 targets.

We focused our analysis on transcription regulators in the mass spectrometry dataset that were also identified by ChIP-Atlas and RegulatorTrail. However, other proteins identified by mass spectrometry may also function as transcription regulators or regulate mRNA abundance through post-transcriptional mechanisms. For example, DDX21, was identified by ChIP-Atlas as a transcription regulator. While complementary evidence was not seen with RegulatorTrail, DDX21 has been reported to have transcriptional regulation activity (Fuller-Pace 2006). Another interesting binding partner identified in our dataset was the cell-surface receptor, EGFR. Nuclear localisation of EGFR has been observed in multiple developmental and malignant cell-types and tissues (Marti et al. 2001; Lin et al. 2001; Psyrri et al. 2005; Li et al. 2008; Wang and Hung 2009; Brand et al. 2011) and EGFR has been reported to directly interact with DNA and function as a transcription factor (Rakowicz-Szulczynska et al. 1986; Lin et al. 2001; Brand et al. 2011). Studies have also shown that EGFR interacts with transcription factors, such as STAT5, STAT3 and E2F1, and regulates gene expression (Lo et al. 2005; Hung et al. 2008). Since GHR is known to cross-talk with the EGFR at the cell surface (Li et al. 2011; Kostopoulou et al. 2017), it is possible that nuclear EGFR interacts with GHR in the nucleus, which is part of our ongoing investigations.

Conway-Campbell et al*.* identified four GHR binding partners using affinity chromatography and tandem mass spectroscopy (Conway-Campbell et al. 2008) and found that the GHR interacted with the transcriptional regulator, coactivator activator protein (CoAA) in a GH-dependent manner, (Conway-Campbell et al. 2008). Although gene transcription was not investigated in this study, CoAA is a potent nuclear receptor coactivator protein that is found to be overexpressed in a wide range of cancers (Sui et al. 2007; Kai 2016), and may contribute to the proliferative actions of nuclear GHR (Conway-Campbell et al. 2008). CoAA was not observed in our mass spectrometry dataset, which could possibly be due to the transient and cell-type specific nature of the protein–protein interactions, or low levels of this protein in the cell. However, consistent with our results, Conway-Campbell et al*.* identified the translational regulator, EF1α as a GHR-binding partner, which was also present in our list of DE proteins identified by co-immunoprecipitation mass spectrometry.

Collectively, our data are consistent with nuclear localisation of the GHR and its interactions with the transcriptional regulator HMGN1 (and potentially SUMO1) forming a novel mechanism through which GHR signalling mediates changes in gene expression. There are a few possible scenarios through which these interactions could manifest following GH stimulation. The lack of an observed interaction between the GHR and HMGN1 in the cytoplasm supports that this interaction occurs after the GHR has translocated into the nucleus. In the nucleus, HMGN1 may either bind to DNA directly while complexed with GHR, or may activate other transcription factors that then mediate changes in gene expression. Earlier studies demonstrated that the GHR is associated with chromatin and likely has transactivating activity (Lobie et al. 1994; Conway-Campbell et al. 2008). Supporting this, Graichen et al. found that nuclear localisation of the extracellular domain of the GHR (GHBP) enhances STAT5-mediated transcription in rat liver cells using a reporter plasmid containing the STAT5 binding element of the serine protease inhibitor 2.1 gene promoter (Graichen et al. 2003). Conway-Campbell et al*.* also reported that the extracellular domain of the GHR acts as a functional transactivation domain in a yeast two-hybrid system. This activity involves amino acid residues 130–246, with serine 226, which is part of the WSXWS cytokine receptor consensus box, being essential for activity (Conway-Campbell et al. 2008). Whether the GHR interacts with specific DNA sequences will be of interest and could be determined by chromatin immunoprecipitation.

In conclusion, this study used two cell lines to confirm previous observations that the GHR translocates into the nucleus in response to GH treatment. HMGN1 was identified as a novel GHR binding partner that co-localises with the GHR in the nucleus after GH treatment. This co-localisation correlated with differential expression of HMGN1 gene targets in the RL95-2 cell-line. Since HMGN1 is a transcriptional regulator that exerts an impact on chromatin conformation by altering the DNA-histone interaction, our results suggest that GH may have a role in epigenetic transcriptional changes. We also found evidence for interaction of the GHR with SUMO1, although were not able to confirm these results through co-immunoprecipitation western blot analysis. Nevertheless, given that GH results in differential expression of SUMO1 gene targets in the RL95-2 cell-line, we suggest that GHR nuclear localisation may also impact gene regulation through SUMO1. These results have potential clinical relevance, with the strong correlation between GHR in the nucleus and increased carcinogenesis (Conway-Campbell et al. 2007) suggesting that prevention of receptor nuclear import may be a therapeutic adjunct. In addition, HMGN1 and SUMO1 might be novel therapeutic targets in pathological conditions associated with aberrant GHR/GH signalling. Further studies investigating the importance of GHR nuclear receptor import and interaction with transcription regulators including HMGN1 and SUMO1 will thus be of interest.

## Supplementary Information

Below is the link to the electronic supplementary material.

Supplementary file1 (XLSX 310 KB)

Supplementary file2 (PDF 729 KB)

## Declarations

### Code availability and data

R scripts and raw data files will be deposited in a data repository and a link provided for the proofs.
