# Supplementary material for: The growth hormone receptor interacts with transcriptional regulator HMGN1 upon GH-induced nuclear translocation
Source: J Cell Commun Signal. 2023 Apr 12;17(3):925–37. doi: 10.1007/s12079-023-00741-2 (PMC10409943; doi:10.1007/s12079-023-00741-2)
Supplement: Supplementary file 2 — Supplementary file2 (PDF 729 KB) [file 12079_2023_741_MOESM2_ESM.pdf]

Supplementary Figures

The growth hormone receptor interacts with transcriptional regulator HMGN1 upon GH-induced nuclear translocation in RL95-2 cells

Lekha Jain[1], Mark H. Vickers[1], Bincy Jacob[2], Martin J. Middleditch[2], Daria A. Chudakova[3], Austen R. D. Ganley[3], Justin M. O'Sullivan[1*], and Jo K Perry[1*]

[1] Liggins Institute, University of Auckland, New Zealand, [2] Faculty of Science, University of Auckland, New Zealand, [3] School of Biological Sciences, University of Auckland

*Co-corresponding authors:

Associate Professor Jo. K. Perry, PhD

Email: j.perry@auckland.ac.nz

Professor Justin M. O'Sullivan, PhD

Email: justin.osullivan@auckland.ac.nz

The Liggins Institute, University of Auckland

85 Park Rd, Private Bag 92019 Auckland 1142, New Zealand

Tel: +64(9) 9237873; Fax: +64(9) 3737497

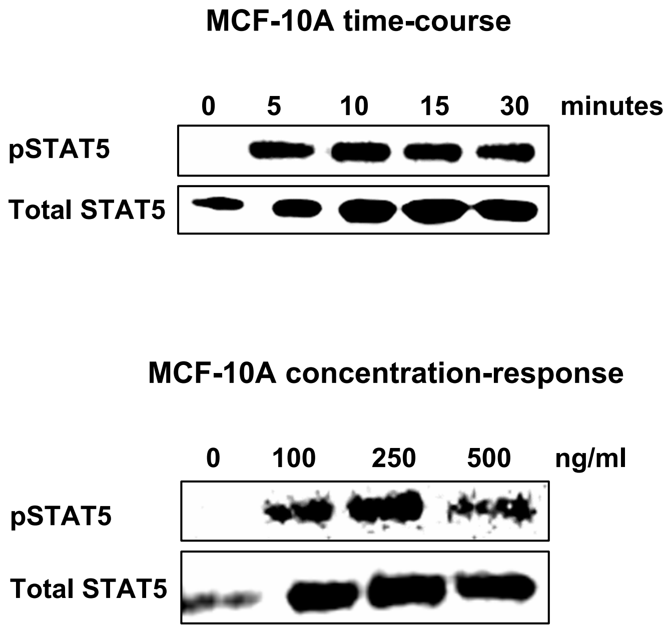

Jain *et al.* Supplementary Figure 1: GH increases STAT5 phosphorylation in MCF-10A cells. (A) GH treatment time-course in MCF-10A cells. Serum-starved MCF-10A cells were treated with 500 ng/ml recombinant human GH for 0, 5, 10, 15 and 30 min. (B) GH treatment concentration response in MCF-10A cells. Serum-starved MCF-10A cells were treated with varying doses of recombinant human GH, 0, 100, 250 and 500 ng/ml for 15 min. Cell lysates were immunoblotted for phosphorylated STAT5 (pSTAT5) and total STAT.

MCF-10A: Anti-GHR_{EC} antibody (Abcam, 89400)

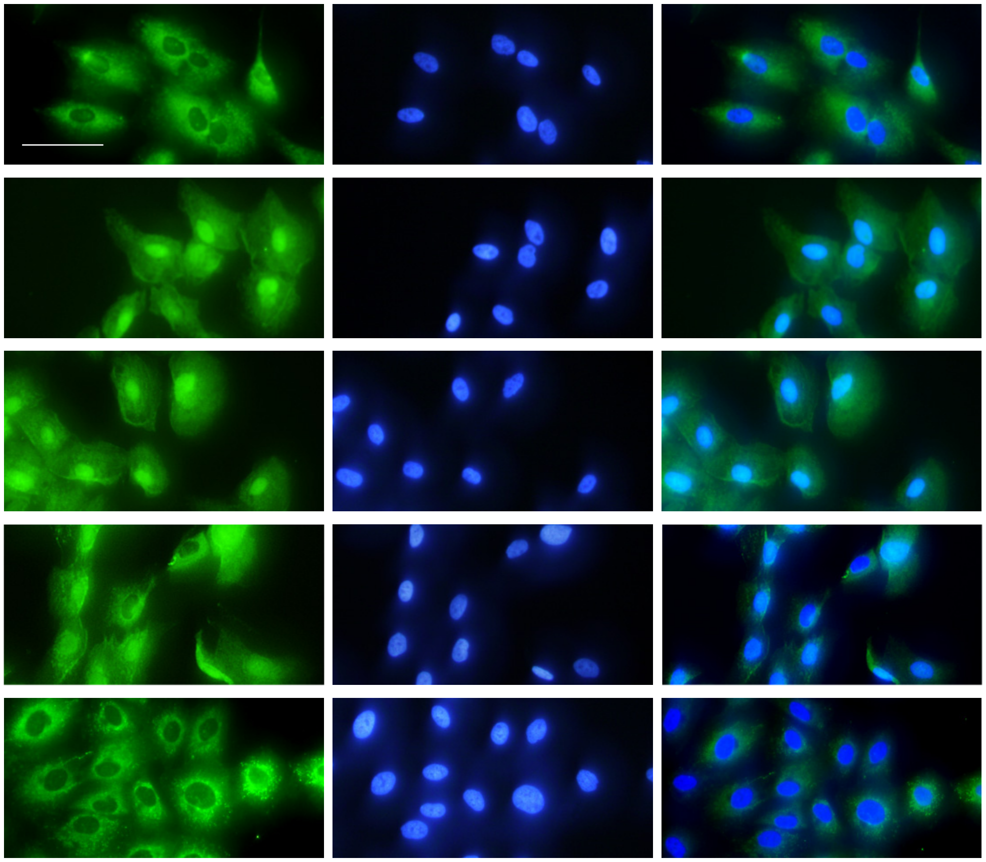

Jain *et al.* Supplementary Figure 2: Nuclear localisation of the GHR in MCF-10A cells. MCF-10A cells were grown on coverslips, serum-starved overnight, and were treated with 250 ng/ml recombinant human GH for 0, 5, 10, 15 and 30 min. Cells were then fixed, permeabilised, blocked and immuno-stained with the anti-GHR_{EC} antibody (Abcam, 89400) and fluorescent secondary antibody. The slides were visualised using fluorescence microscopy. Green (alexa-fluor 488) represents GHR staining and blue (DAPI) is a nuclear stain. The scale bar represents 100 μm.

Anti-GHR_{IC} antibody (sc-137185)

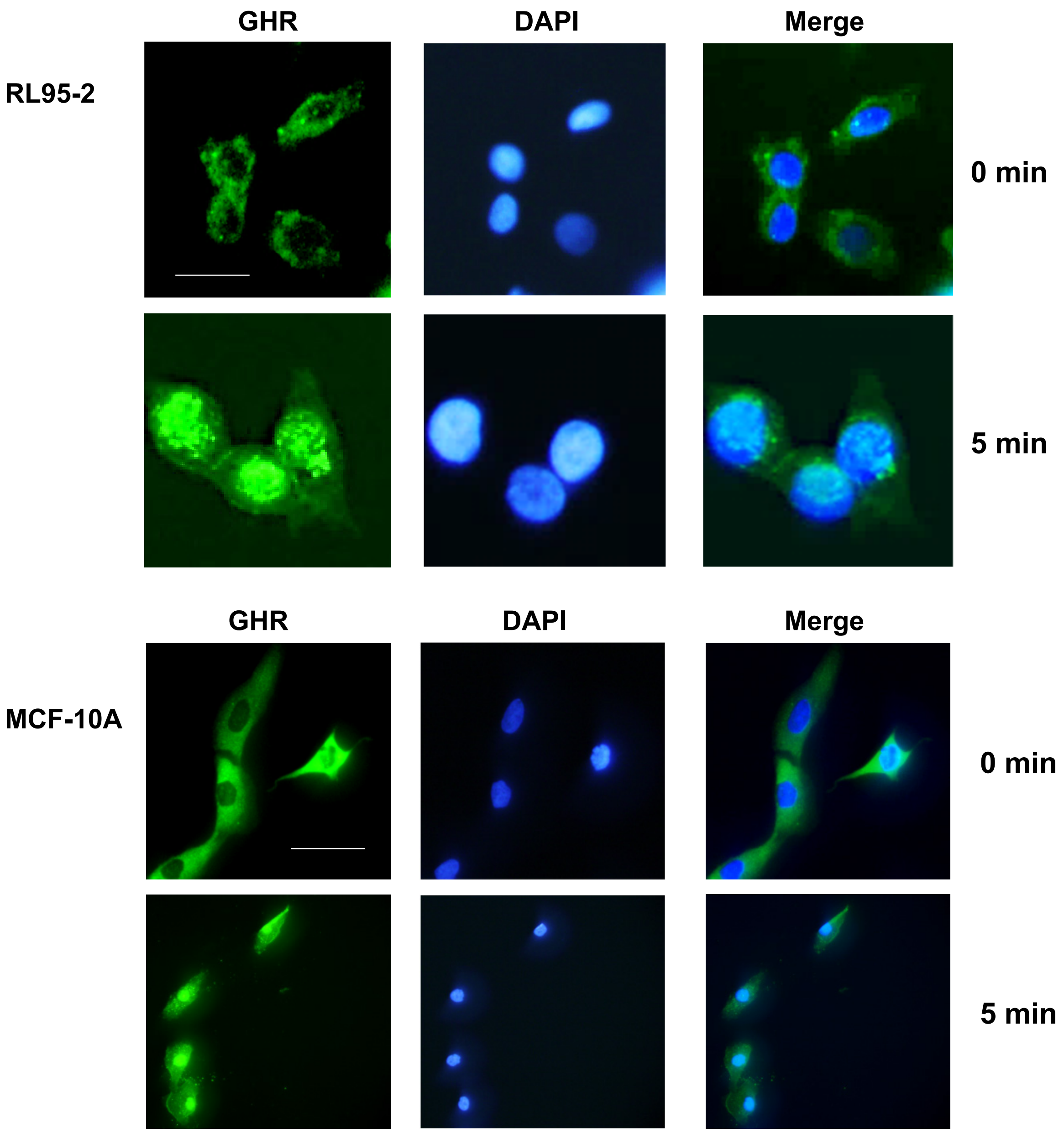

Jain *et al.* Supplementary Figure 3: GHR nuclear localisation in RL95-2 and MCF-10A cells using the anti-GHR_{IC} antibody. Cells were grown on coverslips then serum starved and were treated with 250 ng/ml (MCF-10A) or 500 ng/ml (RL95-2) recombinant human GH for 0 and 5 min. Cells were then fixed, permeabilised, blocked and immuno-stained with the anti-GHR_{IC} antibody (sc-137185) and fluorescent secondary antibody. The slides were visualised using fluorescence microscopy. Green (alexa-fluor 488) represents GHR staining and blue (DAPI) is a nuclear stain. The scale bar represents 100 μm.

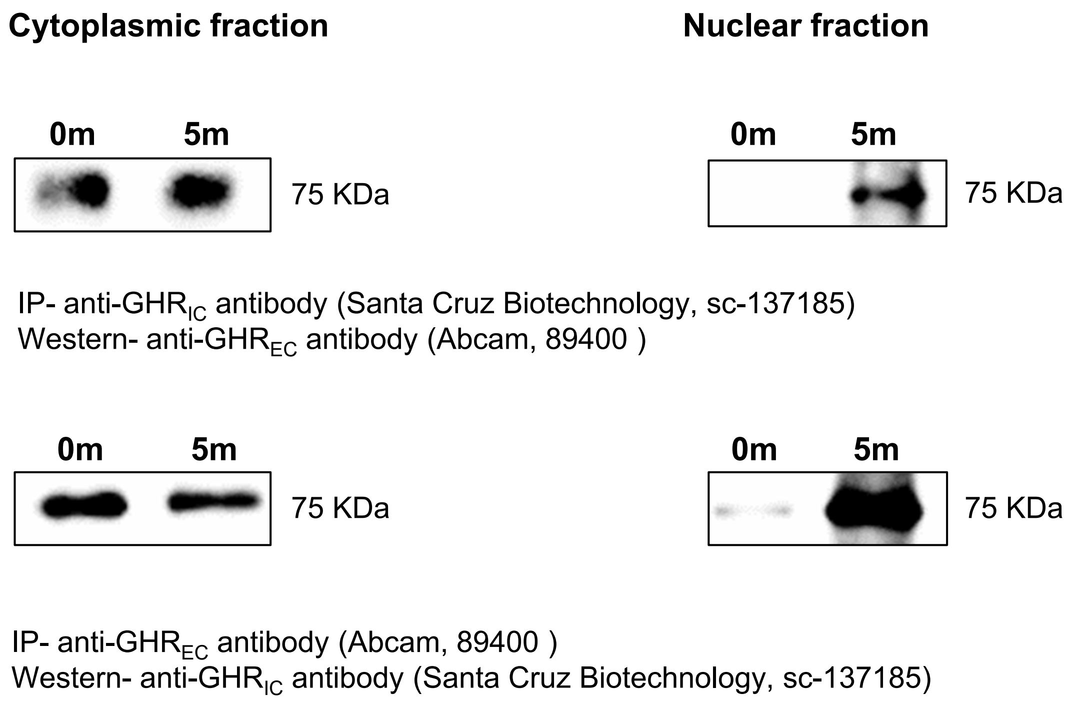

Jain *et al.* Supplementary Figure 4: Serum-starved RL95-2 cells were treated with 500 ng/ml recombinant human GH for 0 and 5 min. Cell lysates were fractionated into cytoplasmic and nuclear fractions, immunoprecipitated and immunoblotted for GHR using the anti-GHR$_{EC}$ or anti-GHR$_{IC}$ antibodies (ab89400 and sc-137185, respectively), as indicated.
